# Next-generation sequencing for identifying a novel/de novo pathogenic variant in a Mexican patient with cystic fibrosis: a case report

**DOI:** 10.1186/s12920-019-0528-1

**Published:** 2019-05-22

**Authors:** Angélica Martínez-Hernández, Julieta Larrosa, Francisco Barajas-Olmos, Humberto García-Ortíz, Elvia C. Mendoza-Caamal, Cecilia Contreras-Cubas, Elaheh Mirzaeicheshmeh, José Luis Lezana, Lorena Orozco

**Affiliations:** 1 Laboratorio de Inmunogenómica y enfermedades metabólicas, Instituto Nacional de Medicina Genómica,SS, Periférico Sur No. 4809, Arenal Tepepan,Tlalpan, 14610. CDMX Mexico City, Mexico; 2 Laboratorio de Inmunogenómica y enfermedades metabólicas, Instituto Nacional de Medicina Genómica, SS, CDMX, Mexico y Posgrado en Ciencias Biológicas, Universidad Nacional Autónoma de México, CDMX Mexico City, Mexico; 3 Area Clínica, Instituto Nacional de Medicina Genómica, SS, CDMX Mexico City, Mexico; 4 Clinica de Fibrosis Quística y Laboratorio de Fisiologia Pulmonar Hospital Infantil de México Federico Gómez. Asociación Mexicana de Fibrosis Quística, A. C, CDMX Mexico City, Mexico

**Keywords:** Cystic fibrosis, Next generation sequencing, P.Trp1089*, P.Glu588*, Novel/de novo variant

## Abstract

**Background:**

Mexico is among the countries showing the highest heterogeneity of *CFTR* variants. However, no de novo variants have previously been reported in Mexican patients with cystic fibrosis (CF).

**Case presentation:**

Here, we report the first case of a novel/de novo variant in a Mexican patient with CF. Our patient was an 8-year-old male who had exhibited the clinical onset of CF at one month of age, with steatorrhea, malabsorption, poor weight gain, anemia, and recurrent respiratory tract infections. Complete sequencing of the *CFTR* gene by next generation sequencing (NGS) revealed two different variants *in trans*, including the previously reported CF-causing variant c.3266G > A (p.Trp1089*, W1089*), that was inherited from the mother, and the novel/de novo *CFTR* variant c.1762G > T (p.Glu588*).

**Conclusion:**

Our results demonstrate the efficiency of targeted NGS for making a rapid and precise diagnosis in patients with clinically suspected CF. This method can enable the provision of accurate genetic counselling, and improve our understanding of the molecular basis of genetic diseases.

## Background

Cystic fibrosis (CF, MIM# 219700) is the most common autosomal recessive disorder among Caucasians. Its incidence varies between different populations, spannig from 1/900 to 1/25,000 or even lower in Eastern populations [1].

Despite the recent instigation of neonatal screening, molecular diagnosis of CF is challenging due to the complex genotype–phenotype relationship, and high genetic heterogeneity [2–4], as found in the Latino American population [5–7]. Since the *CFTR* gene was identified as being responsible for CF [8], over 2000 variants have been detected, with the deletion of phenylalanine at position 508 (c.1521_1523delCTT, p.Phe508del, F508del) being the most frequent worldwide [9]. To date, only nine reported CF cases have involved de novo variants [10–15]. Although Mexico is among the countries showing the highest heterogeneity of *CFTR* pathogenic variants, no de novo variants have previously been reported in Mexican patients.

Here, we report the first case of a novel/de novo variant in a Mexican patient with CF.

## Case presentation

### Clinical phenotype

**Fig. 1:**
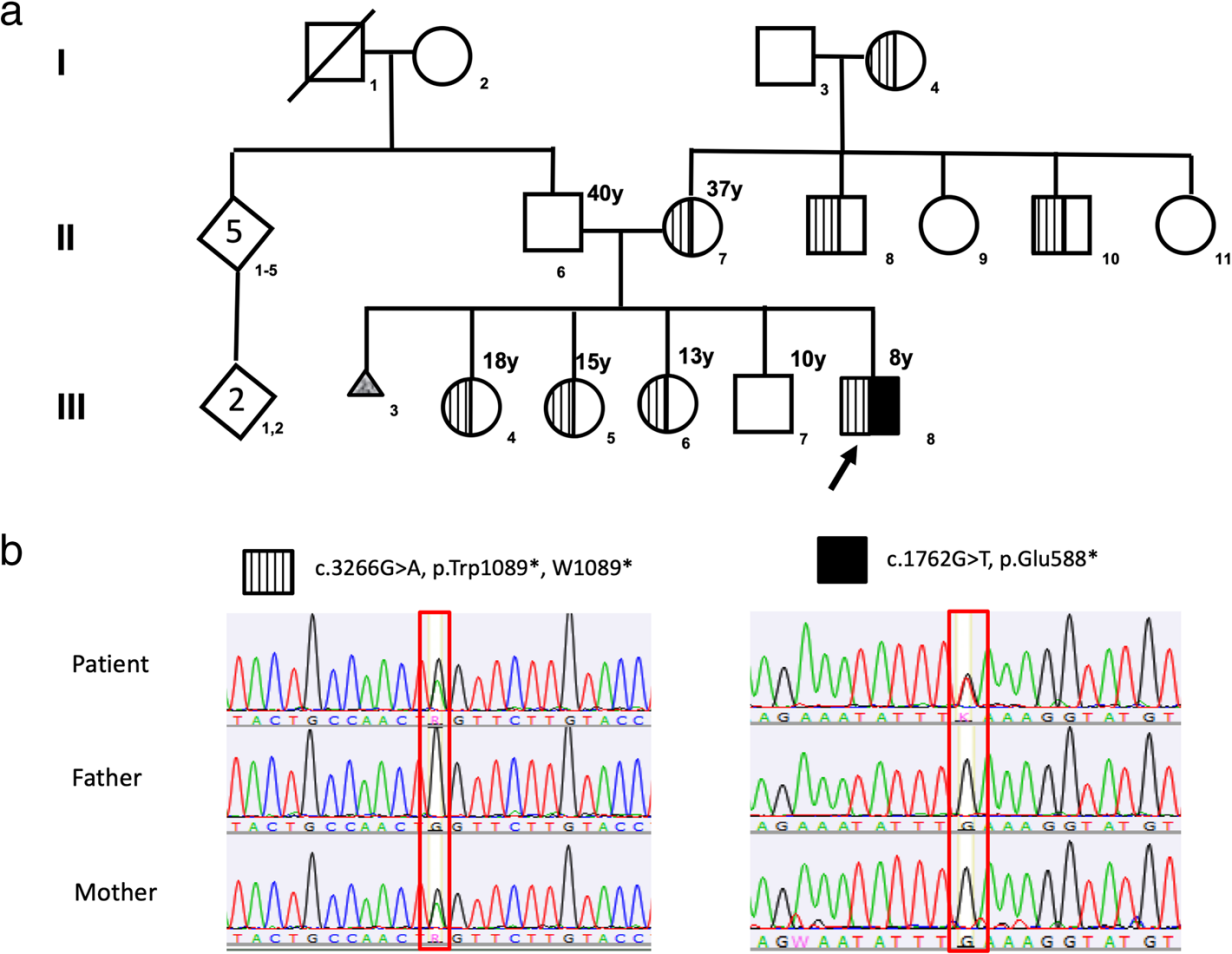
**a** Family pedigree showing the carriers of variants c.3266G > A, p.Trp1089* and c.1762G > T, p.Glu588*, and the patient with both variants (p.Trp1089*/p.Glu588*). **b** Sanger sequencing of the patient, father, and mother. Left, sequence of the variant c.3266G > A, p.Trp1089*. Right, sequence of the variant c.1762G > T, p.Glu588*

An 8-year-old male Mexican patient was referred to our Institution with a diagnosis of CF. He was the fifth child born from healthy non-consanguineous parents, without a family history of the disease (Fig. 1). The mother previously had a spontaneous abortion after 16 weeks gestation by anencephaly. Clinical onset occurred at one month of age with steatorrhea, malabsorption, poor weight gain, and anemia. At 5 months of age, the patient had recurrent respiratory tract infections colonized by *Pseudomonas aeruginosa*. He was diagnosed with CF at 18 months of age, with elevated sweat chloride levels (88, 130, and 129 mmol/l). Currently, the patient’s weight is below the 5th percentile and his height is between the 5th and 10th percentiles.

### Molecular analysis

Using samples from the patient, his parents, and his four siblings, we extracted genomic DNA from peripheral blood lymphocytes using the QIAamp DNA Blood Maxi kit (Qiagen, Valencia, CA, USA) following the manufacturer’s protocol. The index case was screened for the five pathogenic variants that are most frequent in the Mexican population (c.1521_1523delCTT, p.Phe508del; c.1624G > T, p.Gly542*; c.1519_1521delATC, p.Ile507del; c.1646G > A, p.Ser549Asn; and c.3909C > G, p.Asn1303Lys [16–18]), using PCR-mediated site-directed mutagenesis (PSM) as previously reported [19, 20]. Since none of the above-listed variants were identified, we performed complete sequencing of the *CFTR* gene using next generation sequencing (NGS; Illumina HiSeq 2500 sequencer). The NGS data were analyzed using the Genome Analysis Toolkit: UnifiedGenotyper (GATK) and Integrative Genomics Viewer (IGV) software. To predict the functional effect of the variant on the protein, we performed in silico analysis using Variant Effect Predictor (VEP) software [21].

Data collection and molecular analysis were approved by the Research and Ethics Committees at INMEGEN. The parents provided written consent for the child participants, and all children assented.

Our initial screening of the patient revealed none of the pathogenic variants that are most frequent in the Mexican population. Analysis of the completely sequenced *CFTR* gene using GATK and IGV software revealed that the patient carried two different variants *in trans*. One was c.3266G > A (p.Trp1089*, W1089*), which is a previously report CF-causing variation, and the other was the novel variant c.1762G > T, p.Glu588* (https://www.cftr2.org/, [22]). The novel variation involved the change of a G for T in codon 588 of exon 12. Functional effect prediction analysis revealed that this variant induced a premature stop codon (c.1762G > T, p.Glu588*).

Both variants were validated via automated Sanger sequencing. To identify their carrier status, we screened both parents for these variants. The patient’s mother carried only the c.3266G > A (p.Trp1089*, W1089*) variant, while the father carried neither of the variants identified in the patient (Fig. 1). Since the father was not a carrier of any CF variant, paternity was tested using 23 STR genetic markers, which confirmed a paternal probability of > 99.99%. Thus, c.1762G > T, p.Glu588* was determined to be a de novo variant*.* The patient’s three sisters were carriers of the c.3266G > A, p.Trp1089* variant, while his brother showed a wild-type *CFTR* sequence. Additionally, two maternal uncles and the grandmother were also found to carry the c.3266G > A, p.Trp1089 variant.

## Discussion and conclusions

To date, only nine cases of CF caused by de novo pathogenic variants have been reported [10–14]. Most of these variants are single-nucleotide variants, although small and large deletions and partial duplications have also been described [14, 15]. The small number of de novo variants reported in recessive disorders is likely due to the extremely low probability of a de novo variant occurring in combination with the transmission of an allele carrying a pathogenic variation from the other parent. It is also possible that traditional technologies did not enable prior detection of these variants. High-throughput NGS technologies have greatly improved the possibility of identifying rare *CFTR* variants, and of thus further elucidating the genetic heterogeneity of CF [9, 17, 23]. These approaches also dramatically increase the possibility of identifying novel and de novo variants.

In the presently reported case, we identified a novel/de novo variant at position 588, which resulted in a premature stop codon (c.1762G > T, p.Glu588*) in exon 12 of the *CFTR* gene. The patient was an 8-year-old Mexican male who had experienced the clinical onset of CF at one month of age, with pancreatic insufficiency and obstructive lung disease. Compared to inherited variants, de novo variants are probably more deleterious because they have been subjected to less stringent evolutionary selection [24]. However, our patient also harbored the previously reported variant c.3266G > A (p.Trp1089*, W1089*), which is a severe variant that also introduces a stop codon, UAG, at position 1089 in exon 20 [25]. In silico prediction indicated that both identified variants could lead to the production of a truncated protein, decreased translation and elongation accuracy, or production of a final protein that lacks an exon due to exon skipping [26]. Further functional characterization of the novel variant is necessary [3, 27].

The patient carried two class I variants, and thus would be expected to show severe disease, which is in accordance with the observed phenotype. Family analysis revealed that the patient’s mother, three sisters, two uncles, and grandmother were carriers of the c.3266G > A, p.Trp1089* pathogenic variant. However, the c.1762G > T, p.Glu588* variant was not found in the father or in the siblings, and was thus determined to be a novel and de novo variant in our patient with CF. Since our patient harbors two nonsense pathogenic variants, personalized therapy based on the readthrough approach may be appropriate as a future approach [28].

Interestingly, the previously reported novel variants have been located on the paternal chromosome, as in our present case, supporting reports that the male germ line may be more mutagenic [29, 30]. This assumption is related to paternal age at conception, which correlates with the pathogenic variant rate in germline cells. There is presently insufficient information to assess this possibility in CF, since of the nine previously reported CF cases with de novo variants, only two provide information regarding the father’s age (25 and 32 years old). The father of our present patient was 40 years of age.

The present case report demonstrates the efficiency of using targeted NGS to make a rapid and precise diagnosis in patients with clinically suspected CF. This method facilitates the identification of rare or exclusive variants, supporting the provision of accurate genetic counselling and personalized therapy, as well as furthering our understanding of the molecular basis of genetic diseases.

## Abbreviations

CF: Cystic fibrosis
*CFTR*: Cystic fibrosis transmembrane conductance regulator
GATK: Genome Analysis Toolkit
IGV: Integrative Genomics Viewer
INMEGEN: Instituto Nacional de Medicina Genómica
NGS: Next generation sequencing
VEP: Variant Effect Predictor
